# Supplementary material for: Associations between Maternal Cadmium Exposure with Risk of Preterm Birth and Low after Birth Weight Effect of Mediterranean Diet Adherence on Affected Prenatal Outcomes
Source: Toxics. 2020 Oct 20;8(4):90. doi: 10.3390/toxics8040090 (PMC7712046; doi:10.3390/toxics8040090)
Supplement: Supplementary file 1 [file toxics-08-00090-s001.pdf]

Supplementary Materials: Associations between Maternal Cadmium Exposure with Risk of Preterm Birth and Low Birth Weight Effect of Mediterranean Diet Adherence on Affected Prenatal Outcomes

Sarah Gonzalez-Nahm, Kiran Nihlani, John S. House, Rachel L. Maguire, Harlyn G. Skinner and Cathrine Hoyo

Table S1. Regression coefficients and 95% confidence Intervals for the association/relationship between, cadmium exposure (log cd) and birth weight.

Factor	β	95% CI	*p*
Birth weight[a]	77.31	(−688.28, 242.90)	0.8426
Birth weight, no gestational age[d]	−234.11	(−1176.47, 708.24)	0.625
Birth weight, low Med adherence[b]	−126.74	(−907.64, 654.16)	0.7477
Birth weight, high Med adherence[c]	−216.48	(−1257.77,824.81)	0.6804

[a] Adjusted for smoking during pregnancy, pre-pregnancy BMI, gestational age and sex of the infant.[b] Adjusted for smoking during pregnancy, pre-pregnancy BMI, and sex of the infant.[c] Adjusted for smoking during pregnancy, pre-pregnancy BMI, gestational age and sex of the infant, among mothers with a Mediterranean diet score at or below 4.[d] Adjusted for smoking during pregnancy, pre-pregnancy BMI, gestational age and sex of the infant, among mothers with a Mediterranean diet score above 4.

Table S2. Odds ratio and 95% confidence Intervals for the association/relationship between, cadmium exposure (log cd) and preterm birth.

Factor	OR	95% CI	*p*
Preterm birth[a]	0.0093	(0, 1.75)	0.1244
Preterm birth, no birth weight[d]	2.76	(0.09, 48.63)	0.5172
Preterm birth, low Med adherence[b]	0	(0,0.8181)	0.1902
Preterm birth, high Med adherence[c]	0.0001	(0, 50.82)	0.2008

[a] Adjusted for smoking during pregnancy, pre-pregnancy BMI, gestational age and sex of the infant.[b] Adjusted for smoking during pregnancy, pre-pregnancy BMI, and sex of the infant.[c] Adjusted for smoking during pregnancy, pre-pregnancy BMI, gestational age and sex of the infant, among mothers with a Mediterranean diet score at or below 4.[d] Adjusted for smoking during pregnancy, pre-pregnancy BMI, gestational age and sex of the infant, among mothers with a Mediterranean diet score above 4.

Table S3. Regression coefficients and 95% confidence Intervals for the association/relationship between, cadmium exposure (high/low) and 1) Apgar score, 2) Ponderal index.

Factor	β	95% CI	*p*
Apgar score[a]	−0.01	(−0.134 0.12)	0.89
Ponderal index[a]	−0.03	(−0.11, 0.06)	0.51

[a] Adjusted for smoking during pregnancy, pre-pregnancy BMI, gestational age and sex of the infant.
